# An intensity matched comparison of laser- and contact heat evoked potentials

**DOI:** 10.1038/s41598-021-85819-w

**Published:** 2021-03-25

**Authors:** Iara De Schoenmacker, Carson Berry, Jean-Sébastien Blouin, Jan Rosner, Michèle Hubli, Catherine R. Jutzeler, John L. K. Kramer

**Affiliations:** 1 International Collaboration on Repair Discoveries (ICORD), University of British Columbia, Vancouver, BC Canada; 2 Spinal Cord Injury Center, University Hospital Balgrist, University of Zurich, Zurich, Switzerland; 3 School of Kinesiology, University of British Columbia, Vancouver, Canada; 4 Department of Neurology, University Hospital Bern, Inselspital, University of Bern, Bern, Switzerland; 5 Department of Biosystems Science and Engineering, ETH Zurich, Basel, Switzerland; 6 University of British Columbia, Blusson Spinal Cord Centre, 818 West 10th Avenue, Vancouver, BC V5Z 1M9 Canada

**Keywords:** Neuroscience, Physiology

## Abstract

Previous studies comparing laser (LEPs) and contact heat evoked potentials (CHEPs) consistently reported higher amplitudes following laser compared to contact heat stimulation. However, none of the studies matched the perceived pain intensity, questioning if the observed difference in amplitude is due to biophysical differences between the two methods or a mismatch in stimulation intensity. The aims of the current study were twofold: (1) to directly compare the brain potentials induced by intensity matched laser and contact heat stimulation and (2) investigate how capsaicin-induced secondary hyperalgesia modulates LEPs and CHEPs. Twenty-one healthy subjects were recruited and measured at four experimental sessions: (1) CHEPs + sham, (2) LEPs + sham, (3) CHEPs + capsaicin, and (4) LEPs + capsaicin. Baseline (sham) LEPs latency was significantly shorter and amplitude significantly larger compared to CHEPs, even when matched for perceived pain. Neither CHEPs nor LEPs was sensitive enough to detect secondary hyperalgesia. These differences provide evidence that a faster heating rate results in an earlier and more synchronized LEPs than CHEPs. To our knowledge, this was the first study to match perceived intensity of contact heat and laser stimulations, revealing distinct advantages associated with the acquisition of LEPs.

## Introduction

To understand mechanisms underlying the experience of pain, researchers have long sought to characterize the brain’s response to noxious stimulation applied in the periphery^1^. For this purpose, laser evoked potentials (LEPs) have emerged as an important research and clinical tool^2^. Following the success of LEPs, a modern contact heat stimulator, which could be applied to the skin continuously whilst delivering rapid heat pulses (70 °C/s), was developed for the purposes of acquiring nociceptive evoked potentials. This addressed the well-known safety concerns associated with the use of lasers (e.g., skin burns) and the need to wear eye protection^2^.

Despite differences in stimulation parameters, contact heat and laser stimulation generate seemingly similar cortical waveforms with comparable scalp distributions^2–9^. Similarities between methods of acquiring nociceptive evoked potentials extend to brain areas active in response to conventional laser and contact heat stimulation, which, for both, includes the anterior cingulate gyrus and insular cortex^9–13^. LEPs are, however, advocated as a superior approach, owing to a stimulation that is “purely nociceptive”, which penetrates the skin to directly activate nociceptors^5^.

Several published studies have examined both LEPs and CHEPs in the same healthy subjects, allowing for a direct comparison of outcomes between stimulation modalities^5,14–19^. In addition to shorter latencies, contralateral and vertex potentials are consistently larger in response to laser stimulation and, in the case of the N1 waveform, more reliably acquired^5^. This is beneficial for clinical applications, where various pathologies in the spinal cord and brain may ultimately render potentials small and more difficult to acquire^20,21^. A limitation of previous studies, however, is the lack of control for how subjects perceived noxious stimulation. This is problematic because conventional contact heat stimulators are bound by upper temperature limits (nominally 54 °C), generally producing lower pain ratings compared to laser stimuli^5,19^. For example, in a seminal study comparing LEPs and CHEPs^5^ pain ratings were approximately 1-point less on a numeric rating scale after contact heat compared to laser stimulation. Given that more intensely perceived stimuli evoke larger potential amplitudes for both CHEPs and LEPs^9^, this raises the question of whether differences between CHEPs and LEPs is attributable to this methodological limitation. An understanding of the real difference between CHEPs and LEPs is needed to guide the development of novel contact heat stimulators^22^.

Chief among LEPs and CHEPs clinical applications is the detection of peripheral and central pathology related to neuropathic pain^2,23^. As an experimental model, the topical application of capsaicin, a potent agonist of transient receptor potential cation channel subfamily V member 1 (TRPV-1), results in distinct areas of primary and secondary hyperalgesia corresponding with mechanisms underlying peripheral and central sensitization, respectively^24–26^. Secondary hyperalgesia is evidenced in the surrounding, non-treated skin area as hyperalgesia to mechanical punctate stimulation, which is thought to reflect increased activity of second order neurons in the dorsal horn^27,28^. To detect peripheral and central sensitization, studies applying contact heat and laser stimulations have reported variable and conflicting changes in vertex and contralateral potentials following stimulation in the primary and secondary areas^13,29–34^. A design limitation of previous studies is that none included a sham condition. Moreover, noxious stimulation was applied in sequence before and after the application of capsaicin. These issues are important to consider because the expectation that capsaicin modulates perception could have profound effects on evoked potentials^35^, and subtle changes could be a function of repetitive testing in the same stimulation area on the same day^9^. Furthermore, none have applied both laser and contact heat stimulations in the same subjects to determine if one method is more suitable than the other to detect sensitization. This is of critical importance towards establishing stimulation parameters that accurately and sensitively detect peripheral and central sensitization in patients with chronic pain^36^.

To comprehensively compare LEPs and CHEPs, we designed a study that carefully matched laser and contact heat stimulation based on subject ratings. In separate experimental sessions, CHEPs and LEPs were acquired following noxious stimulation applied to the volar forearm, after the topical application of sham or capsaicin cream. Our primary analysis aimed to address differences in CHEPs and LEPs latencies and amplitudes at rest (i.e., sham) and in the secondary area of hyperalgesia (i.e., after topical application of capsaicin). We hypothesized shorter latencies and larger amplitudes of LEPs than CHEPs. Given variability in previous studies regarding the effects of central sensitization on nociceptive evoked potentials, our aim to examine CHEPs and LEPs in the area of secondary hyperalgesia was exploratory.

## Methods

### Subjects

A total of 21 healthy subjects (13 males, 8 females) aged between 19 and 45 years were recruited. Subjects taking medication other than birth control medication were excluded. Written informed consent was obtained from every subject prior to participation. The University of British Columbia (UBC) ethics board approved the study (H18-01259) and all experimental procedures were in accordance with the Declarations of Helsinki.

### Study design

**Figure 1:**
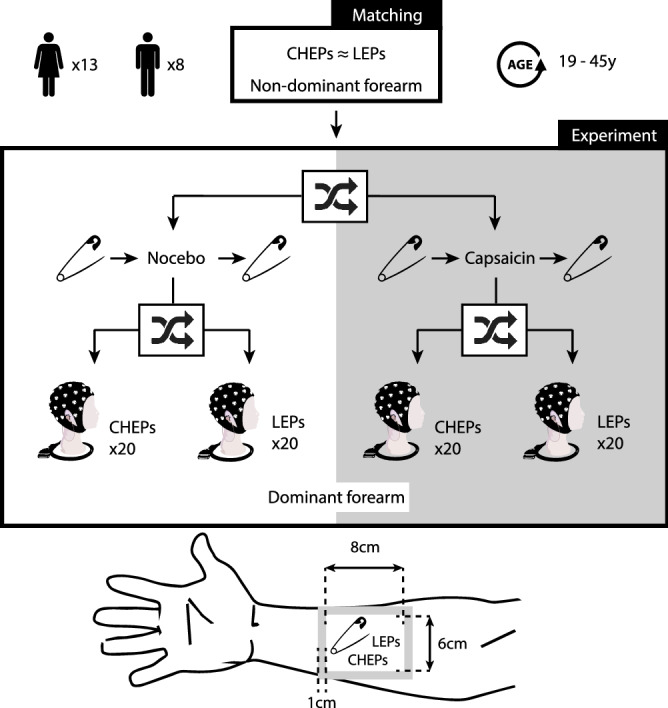
Study design. Subjects first underwent an intensity-matching protocol on the non-dominant forearm (upper box). Using a stair-case paradigm, CHEPs and LEPs were matched for stimulation intensity based on individual perception. Subsequently, subjects were randomized to the capsaicin (white background) or sham (gray background) group. Both experimental groups started with the assessment of pain sensation using pinprick stimulation within the confirmed secondary hyperalgesia area on the dominant volar forearm. After 30 min of topical application (sham or capsaicin), the pinprick protocol was repeated and then 20 laser or contact heat stimulations were applied. Brain responses to contact heat and laser stimulations applied in the secondary hyperalgesia area were recorded with a 32-channel EEG set up. Subjects were exposed to all four conditions in a randomized order. The volar forearm at the bottom is an illustration of the 1 ml capsaicin or sham cream application (gray perimeter with 1 cm width). The measured pinprick, laser and contact heat site is the inner square with the size of 6 × 8 cm (expected area of secondary hyperalgesia) and is centered to the forearm (wrist to elbow).

The study design is illustrated in Figure 1. Each subject participated in all four single-blinded experimental sessions, which involved: (1) CHEPs + sham, (2) LEPs + sham, (3) CHEPs + capsaicin, and 4) LEPs + capsaicin. All sessions were one week apart and performed in randomized order. At the onset of each experimental session, 11 contact heat stimuli were applied on the non-dominant volar forearm to familiarize subjects to stimulation. Subjects were asked to rate each stimulus using a numeric rating scale (NRS) ranging from 0 to 10 (i.e., 0 being no pain, 1 the first sensation of burning or stinging and 10 worst pain imaginable). The first stimulus was discarded from the 11 familiarization contact heat stimuli due to novelty and surprise^37^. To avoid sensitizing the peripheral area, a break of 5 min was introduced between the familiarization period with CHEPs stimulation and LEPs intensity matching. In a next step, mechanical pain sensitivity on the dominant volar forearm was measured using quantitative pinprick stimulations before and after capsaicin or sham cream application. Subjects were blinded in terms of cream application and only instructed that the cream could potentially result in sensations of itching or burning. The mechanical punctate sensitivity was examined to confirm capsaicin (but not sham) resulted in an area of secondary hyperalgesia. Finally, either laser or contact heat was applied in the same area as the pinprick protocol within the confirmed secondary hyperalgesia for the acquisition of evoked potentials.

### Pin prick protocol and capsaicin application

The stimulation area for pinprick, laser, and contact heat was a 6 × 8 cm rectangle centered in the middle of the dominant volar forearm (Fig. 1). In this area, ratings of perceived pain intensity to seven differently weighted pinpricks (i.e., 8, 16, 32, 64, 128, 256, and 512mN) were evaluated (http://www.mrc-systems.de/en/products/pinprick). Each weighted pinprick was used five times in a randomized order. One milliliter of either capsaicin (0.075% Haenseler AG, Switzerland) or sham cream (commercially available moisturizer https://www.aveeno.com/products/daily-moisturizing-lotion) was applied on a 1 cm perimeter around the stimulation area (see Fig. 1). For each subject and experimental session, the average of all pain ratings was calculated (pre- and post-cream separate) as an overall measure of changes in sensitivity.

### LEPs matching protocol

The intensity of laser stimulation was matched to that of contact heat. This was done because we anticipated that the highest temperature contact heat stimulation (i.e., nominally, 54 °C)^22^ would result in lower ratings than the highest laser stimulation intensity. The parameters were thereby fixed measuring CHEPs and adjusted in terms of stimulation intensity when stimulating with the laser.

A double random staircase (DRS) method^38^ was used to match the perceived intensity of the laser pain to the perception of the contact heat pain. Briefly, DRS consisted of two concurrent, randomly interspersed staircases (10 stimuli each) around the average of the initial 10 contact heat pain ratings. Staircases started at 1.5 J and incremented by 0.5 J per stimulus if subjects did not feel pain or incremented and decremented by 0.25 J when the laser pain was lower and higher than the contact heat pain, respectively. Once a staircase began oscillating around the average contact heat pain score, the median laser stimulation intensities and pain ratings from each staircase were calculated separately. Finally, the average of the two median staircase values (i.e., one median laser stimulation intensity for each staircase) was used as the matched laser stimulation intensity for subsequent acquisition of LEPs. In case the average laser pain score of the two NRS medians was lower than the average contact heat pain score, an intensity of 0.25 J was added to the matched laser intensity. This decision was based on our experience that participants tended to underestimated laser stimulation intensity. An example of the procedure can be found in the supplementary information (Fig. S.1).

### CHEPs and LEPs

Subjects were lying in a supine position with their eyes open, fixating on a point on the ceiling. In each experimental session, subjects underwent 20 stimulations with either contact heat (PATHWAY, Medoc Advanced Medical System, Israel) or laser (STIMUL 1340 Nd: Yap laser, Electronic Engineering, Italy). The contact heat baseline temperature was set at 35 °C, and the thermode (27 mm diameter) ramped to 52 °C with a nominal heating rate of 70 °C/s and retuned to baseline with a cooling rate of 40 °C/s^21^. 20 laser stimulations were delivered with 5 ms pulse and diameter of 5 mm. Both stimulators relayed a trigger at the stimulation onset. Contact heat and laser stimulations were delivered with an inter-stimulus interval ranging between 8 to 12 s. After each stimulation the device was moved within the 6 × 8 cm area to reduce receptor fatigue or habituation^8^. Perceived pain was rated by the subject after each stimulation using a 0–10 NRS.

### EEG recording

In accordance with the international 10–20 system, 32 EEG electrodes (ANT Waveguard 64, Welbergweg 74 7556 PE Hengelo, Netherlands, available from: https://www.ant-neuro.com/products/waveguard_original) were placed on the scalp^39^. All impedances were kept below 15kΩ. Two reference electrodes were placed behind the ears and a non-reference ground was placed on the wrist of the non-dominant arm. The EEG signal was digitized at 4000 Hz (TMSi Refa 72, Zutphenstraat 57, 7575 EJ Oldenzaal, The Netherlands, available from: https://www.tmsi.com/products/refa/) and recorded with modified software available from (https://www.tmsi.com/forum/tmsi-matlab-interface/1-download-tmsi-matlab-interface) in MATLAB (R2015b, Mathworks, Inc., Nattick, USA). EEG data were processed using the EEGlab toolbox (V.14_1_1b, SCCN)^40^. The continuous EEG data were bandpass-filtered offline between 1 and 40 Hz, using a first order Hamming windowed finite impulse response (FIR) filter^41^ and baseline corrected. The data were divided into epochs, starting from one second pre-stimulus, to two seconds post-stimulus. Eye blinks and recording artifact components were removed using independent component analysis, informed by conservative use of the autocorrelation and focal options of the SASICA toolbox^42^.

The signal to noise ratio (SNR) was calculated in decibels (dB) for each recording session by comparing the root mean square voltage at the Cz electrode of the pre-trigger window to the root mean square voltage in the time region of interest, which was 150–550 ms post-trigger for LEPs and 300–650 ms post-trigger for CHEPs^43^. We investigated N1 as well as the N2 and P2 waveforms. Physiological unrealistic LEPs and CHEPs related to artefact or noise dominating signals (SNR below 3 dB) were excluded. Two independent reviewers evaluted evoked potential to mark N1, N2, and P2 waveforms. This was done on a visual basis after artifact removal. At a minimum, the waveform needed to be greater than the SNR of a 500 ms pre-trigger interval. For N1, the latency of negative potential must have had proceeded the onset of N2. The latencies and amplitudes of the N2 and P2 signals were determined from the Cz electrode referenced to linked ear electrodes (behind helix). N1 was recorded from the electrode (C3 or C4) contralateral to the stimulated side and referenced to Fz. For absent LEPs and CHEPs, 0 µV was assigned for amplitude to allow for inclusion in our statistical analysis.

### Statistical analysis

All statistical analyses were performed using R Studio (R version 3.5.1 for PC). Descriptive analyses were determined for the demographics, NRS, and LEPs and CHEPs outcomes (mean, + /− standard deviation). To determine the effectiveness of our intensity matching protocol, average NRS following contact heat and laser stimulations was correlated using Pearson correlation. Additionally, matching of contact heat and laser pain was tested using an intraclass correlation coefficient (ICC) with a two-way average agreement model. To compare baseline CHEPs and LEPs (sham condition), a paired t-test was used on the parameters of interest (amplitudes and latencies). To test if capsaicin induces mechanical hyperalgesia (i.e., increased sensitivity to mechanical punctate stimuli), the difference in pre-post sham and pre-post capsaicin pain was examined (CHEPs and LEPs session separately). The differences in condition (sham and capsaicin) of mechanical hyperalgesia, CHEPs and LEPs were analyzed using a paired t-test. Statistical significance was set at α = 0.05. To compare CHEPs and LEPs Bonferroni corrections were applied to account for multiple comparisons (N1, N2 and P2 latency and amplitude and N2P2 amplitude). The adjusted level of significance was thereby α = 0.007. EEG processing pipelines can be found on Kramer lab GITHUB repository (https://github.com/kramerlabvancouver/EEG_work).

## Results

### Subjects

Of the 21 subjects recruited, one had to be excluded due to not tolerating the familiarization contact heat stimulation. Table S.1 in the supplementary information summarizes relevant study data.

Two additional subjects dropped out and did not complete all four experimental sessions (i.e., C08 and C09, see Table S.1) due to time reasons. The matched laser intensity is missing as a consequence of technical issues for one session in two subjects (i.e., C14 and C15, see Table S.1).

### Baseline measurements

#### Contact heat and laser evoked potentials

**Figure 2:**
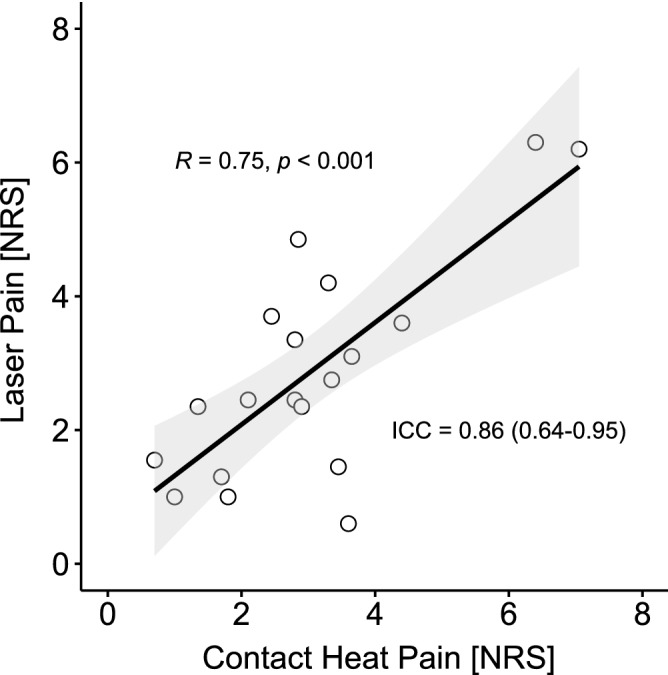
Perceived pain matching. Illustrated is the correlation between the contact heat (x-axis) and laser (y-axis) perceived pain as well as the intraclass correlation coefficient (ICC).

Figure 2 illustrates the correlation of contact heat and laser perceived pain, as well as the ICC.

**Figure 3:**
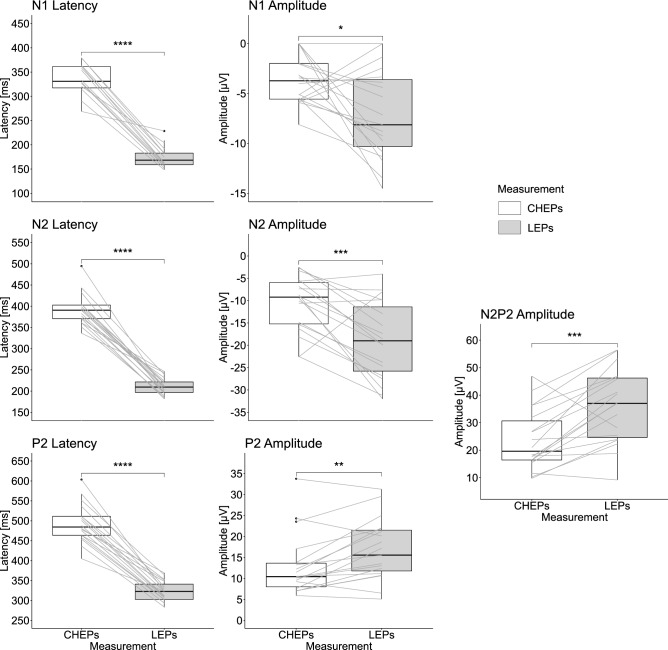
CHEPs and LEPs baseline comparison. The measurement illustrates on the x-axis, CHEPs in white and LEPs in gray. The y-axis illustrates the LEPs and CHEPs parameters (latency or amplitude). Subjects’ responses of both methods are connected by a grey line.

The LEPs and CHEPs of one subject had to be excluded due to low SNR (SNR threshold >  = 3 dB, i.e., C08, see Table  S.1). The remaining 19 subjects had detectable N2P2 waveforms following both contact heat and laser stimulation in the sham and capsaicin sessions. N1 waveforms were less reliably detected (SNR threshold >  = 3 dB, CHEPs sham: n = 14, CHEPs capsaicin n = 13, LEPs sham n = 17, LEPs capsaicin n = 15). There was no correlation between CHEP outcomes (e.g., N1/N2/P2 amplitude) and NRS or LEP outcomes and NRS (all p > 0.05). LEP waveforms were significantly shorter and larger compared to CHEPs (see Table 1 and Fig. 3).

**Table 1:** CHEPs and LEPs baseline parameter comparison.

	CHEPs (mean ± sd)	LEPs (mean ± sd)	t score	df	p-value
N1 latency	336 ± 33 ms	175 ± 23 ms	13.52	12	< 0.001
N1 amplitude	− 3.6 ± 2.5 µV	− 7.1 ± 4.5 µV	2.67	17	0.016
N2 latency	392 ± 37 ms	211 ± 19 ms	17.99	17	< 0.001
N2 amplitude	− 10.6 ± 6.3 µV	− 18.9 ± 8.6 µV	4.09	17	< 0.001
P2 latency	494 ± 49 ms	322 ± 26 ms	15.59	17	< 0.001
P2 amplitude	13.0 ± 7.4 µV	16.8 ± 7.2 µV	− 3.58	17	0.002
N2P2 amplitude	23.6 ± 11.0 µV	35.7 ± 13.3 µV	− 4.47	17	< 0.001

CHEPs: contact heat evoked potentials; LEPs: laser evoked potentials; df: degree of freedom; sd: standard deviation.

### Pain modulation

#### Mechanical and thermal hyperalgesia after capsaicin application

**Figure 4:**
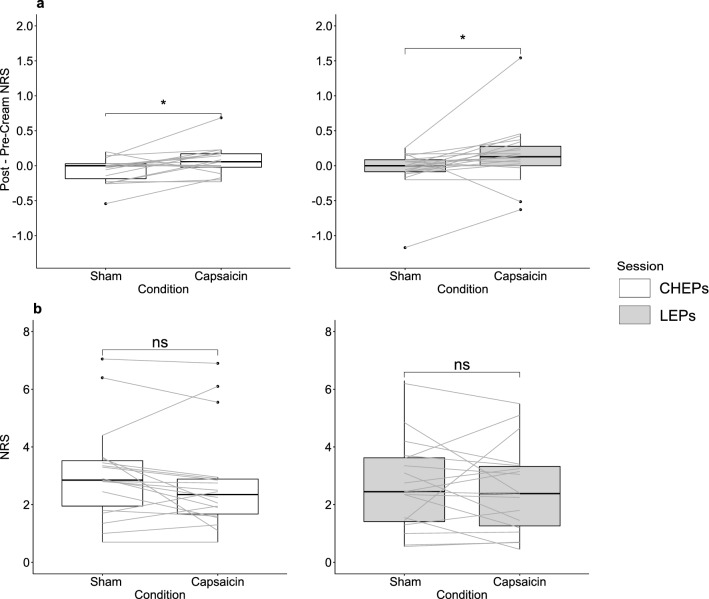
Measure of Mechanical and Thermal Hyperalgesia. Comparison of the sham and capsaicin condition for (row **a**) mechanical and (row **b**) thermal pain rating for the CHEPs (white background) and LEPs session (grey background) separately. The condition (sham and capsaicin) is illustrated on the x-axis. In (row **a**) the y-axis illustrates the difference in overall pain rating, which is calculated by subtracting the pre-cream from the post-cream pain. In (row **b**) the y-axis illustrates average pain rating during the sessions. Subjects’ responses of both conditions are connected by a grey line.

For both experimental sessions (CHEPs and LEPs), capsaicin cream induced secondary mechanical hyperalgesia. This was evidenced as significantly increased perceived sensation to pinprick stimulation within the area adjacent to capsaicin compared to sham (Table 2, Fig. 4a). There was no change in thermal pain sensation for either CHEPs or LEPs after capsaicin application (Table 2, Fig. 4b).

**Table 2:** Mechanical and thermal hyperalgesia.

	Sham (mean ± sd)	Capsaicin (mean ± sd)	t score	df	p-value
Post–pre-cream pin prick pain (CHEPs session)	− 0.1 ± 0.2NRS	0.1 ± 0.2NRS	− 2.85	17	0.011
Post–pre-cream pin prick pain (LEPs session)	0.0 ± 0.3NRS	0.2 ± 0.4NRS	− 2.36	19	0.029
Contact heat pain	3.0 ± 1.6NRS	2.7 ± 1.7NRS	1.59	17	0.131
Laser pain	2.8 ± 1.7NRS	2.5 ± 1.5NRS	0.38	17	0.708

CHEPs: contact heat evoked potentials; LEPs: laser evoked potentials; df: degree of freedom; sd: standard deviation.

#### Contact heat and laser evoked potentials after central sensitization

**Figure 5:**
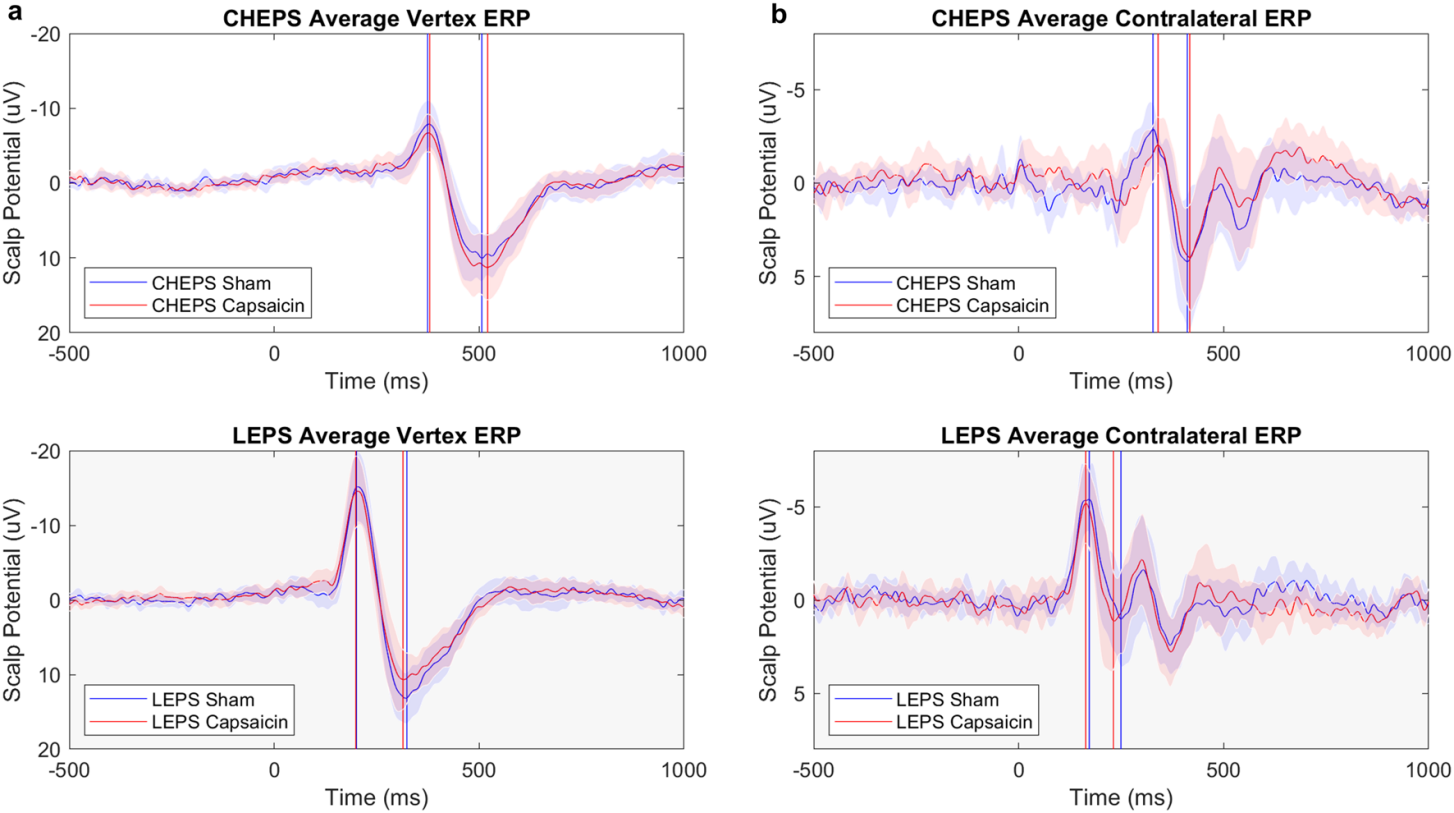
CHEPs and LEPs Grand Average. Illustrated are the grand average potentials measured at (column **a**) the vertex (N2P2 waveform) and (column **b**) contralateral electrodes C3 or C4 (N1 waveform). CHEPs are indicated with a white background and LEPs with a grey background. The blue lines illustrate the grand average of the sham condition and the red line the grand average of the capsaicin condition. Included are the 95% confidence intervals, shown with a less dense blue and red area. N2 occurs around 377 ms for CHEPs and 205 ms for LEPs. P2 occurs around 507 ms for CHEPs and 321 ms for LEPs. N = 19. N1 occurs around 327 ms and 160 ms for CHEPs and LEPs, respectively.

**Figure 6:**
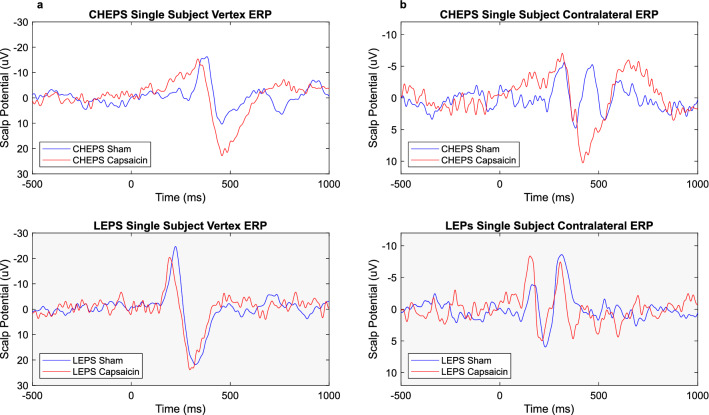
CHEPs and LEPs Single Subject Average. Illustrated are the average potentials measured at (column **a**) the vertex (N2P2 waveform) and (column **b**) contralateral electrodes C3 (N1 waveform) of subject C14. CHEPs are indicated with a white background and LEPs with a grey background. The blue lines illustrate the grand average of the sham condition and the red line the grand average of the capsaicin condition.

There was no difference in latency and amplitude (Tables 3 and 4) after capsaicin application for either LEPs or CHEPs. Grand averages for LEPs and CHEPs (N2P2 and N1) are shown in Figure 5. Figure 6 shows an example of a single subject (C14) recording of CHEPs and LEPs with both conditions (sham and capsaicin).

**Table 3:** CHEPS sham and capsaicin comparison.

CHEPs	Sham (mean ± sd)	Capsaicin (mean ± sd)	t score	df	p-value
N1 latency	336 ± 33 ms	346 ± 30.7 ms	− 0.29	10	0.778
N1 amplitude	− 3.6 ± 2.5 µV	− 3.3 ± 2.8 µV	− 0.39	17	0.703
N2 latency	392 ± 37 ms	387 ± 32 ms	0.81	17	0.431
N2 amplitude	− 10.6 ± 6.3 µV	− 10.2 ± 4.6 µV	− 0.37	17	0.719
P2 latency	494 ± 49 ms	493 ± 45 ms	0.11	17	0.914
P2 amplitude	13.0 ± 7.4 µV	14.8 ± 8.3 µV	− 1.34	17	0.197
N2P2 amplitude	23.6 ± 11.0 µV	25.1 ± 11.5 µV	− 0.97	17	0.347

CHEPs: contact heat evoked potentials; df: degree of freedom; sd: standard deviation.

**Table 4:** LEPS sham and capsaicin comparison.

LEPs	Sham (mean ± sd)	Capsaicin (mean ± sd)	t score	df	p-value
N1 latency	175 ± 23 ms	179 ± 35 ms	− 0.71	14	0.492
N1 amplitude	− 7.1 ± 4.5 µV	− 5.9 ± 5.4 µV	− 0.84	18	0.413
N2 latency	211 ± 19 ms	216 ± 26 ms	− 0.89	18	0.388
N2 amplitude	− 18.9 ± 8.6 µV	− 18.6 ± 9.4 µV	− 0.26	18	0.799
P2 latency	322 ± 26 ms	323 ± 33 ms	− 0.19	18	0.853
P2 amplitude	16.8 ± 7.2 µV	15.7 ± 8.5 µV	0.81	18	0.428
N2P2 amplitude	35.7 ± 13.3 µV	34.3 ± 16.2 µV	0.67	18	0.512

LEPs: laser evoked potentials; df: degree of freedom; sd: standard deviation.

## Discussion

The goal of this study was twofold. First, we aimed to examined matched-intensity cortical evoked potentials in response to contact heat and laser stimulation. After carefully matching for perceived stimulus intensity (ICC > 0.8), contralateral and vertex potentials evoked by laser were shorter latency and larger amplitude compared to corresponding potentials evoked by contact heat. Our second aim evaluated the extent that LEPs and CHEPs revealed evidence of central sensitization. The study design addressed a specific limitation of previous studies that did not incorporate a sham condition, thus not controlling for potential sham effects. In an area of secondary hyperalgesia caused by the topical application of capsaicin, neither LEPs nor CHEPs yielded evidence of central sensitization.

### Comparison of LEPs and CHEPs at matched perceived pain intensities

For all cortical waveforms, latencies were significantly shorter following laser stimulation compared to contact heat. This included for the earliest, contralateral N1 potential, which reflects the most direct cortical measure of noxious afferent processing^44^. Shorter latency LEPs are not surprising in light of instantaneous activation of nociceptors, which recruit A-delta afferents faster than contact heat stimulation^5^. Similar observations have been reported for a matched comparison of CO2 or Nd:YAP laser stimulation; the latter penetrating the skin to better activate nociceptors, yielding faster rise times and larger evoked potentials^5^. In our study, LEPs were also generally of larger amplitude than those arising from stimulation with contact heat. This effect was observed even though perception was meticulously matched between the two stimulation modalities. While this generally corresponds with previous studies acquiring both LEPs and CHEPs, ours was the first to explicitly control for differences in pain perception and limit the confounding effects of stimulating multiple times in a single experimental session. As perceived intensity is the main determinant of stimulus saliency^45^, controlling the former through intensity matching renders contact heat and laser stimulations equally salient. The larger amplitudes associated with laser stimulation thus demonstrate the importance of synchronous activation in the periphery for the acquisition of N1, N2, and P2 waveforms, which cannot be overcome by greater stimulation area (i.e., contact heat) and which are unlikely confounded by differences in stimulation saliency. Overall, this indicates the need for faster and more direct recruitment of peripheral afferents to improve the acquisition of CHEPs, which may be possible with advances in contact heat stimulation technology (i.e., increased rates of temperature change)^22^.

### Impact of central sensitization on contact heat and laser evoked potentials

Numerous studies have applied topical capsaicin as a means of experimentally inducing sensitization and examining the impact on nociceptive evoked potentials^10,13,29–31,46–48^. Generally speaking, primary and secondary areas of hyperalgesia are reportedly accompanied by reduced LEPs amplitudes following stimulation in corresponding area^13,29,30^. This represents somewhat of a paradox, insofar as reduced amplitudes are generally considered evidence of desensitization (i.e., opposite to that expected in relation to sensitization). In line with the concept of lowering the threshold of nociceptor activation, multiple studies demonstrate reduced CHEPs latencies following stimulation in the primary area of hyperalgesia^23,31,34^. This, in principle, makes sense for contact heat stimulation, in that temperature increases from below a critical threshold to activate nociceptors earlier after sensitization with capsaicin, and points to a potential advantage compared to LEPs.

Regardless of the stimulation modality, we observed no effect of capsaicin-induced central sensitization on evoked potential latency and amplitude parameters. Accompanying null effects, we also detected no measurable change in perception to laser or contact heat stimulation (i.e., ratings remained constant between sham and experimental conditions). Critical to the aim of our study (i.e., to measure the effects of central sensitization), we did, however, observe a significant degree of mechanical hyperalgesia to pinprick after capsaicin application. This is consistent with secondary hyperalgesia being accompanied by increased sensitivity to mechanical but not heat stimulation^26,49–51^—observations that are supported by sensitization of pinprick evoked potentials^22^. Overall, our results suggest that secondary hyperalgesia does not involve the sensitization of nociceptors activated in response to laser or contact heat stimulation (i.e., type II A-delta mechano-heat)^51^.

A number of factors may explain the divergence in our results compared to others applying topical capsaicin to induce central and peripheral sensitization. One possibility is differences in study design. In general, previous studies have adopted varying types of control and acquired nociceptive evoked potentials before and after the topical application of capsaicin. Those taking a within subject approach have either assumed that repetitive laser or contact heat stimulation has no or minimal effects on evoked potential outcomes^13,29,31,46,48^ or incorporated heterotopic stimulation as a control (e.g., contralateral to dermatome where capsaicin was applied). Both approaches are problematic because multiple bouts of stimulation and acquisition of nociceptive evoked potentials on the same day can result in reduced amplitudes and delayed latencies due to unrelated mechanisms (e.g., habituation)^52^. A parallel approach, incorporating a separate control group unexposed to capsaicin, provides an estimate of normally occurring habituation but does so in a distinct cohort of subjects. This is problematic because sample sizes have been generally small (e.g., n = 15)^33^, which has potentially lead to underpowered observations and type I error.

Our design utilized a within subject, sham control, with subjects being examined on separate days. Only one acquisition of CHEPs or LEPs was performed in each experimental session – a design feature that made the study more challenging (i.e., each subject participated on 4 separate session, which inevitably resulted in subjects dropping out). Because LEPs and CHEPs parameters are generally reliable within subjects between experimental sessions^2,53,54^, significant changes between sham and capsaicin can be attributed to mechanisms underlying secondary hyperalgesia, thus providing an unbiased estimate of central sensitization. In the absence of such a change, we conclude that LEPs and CHEPs are insensitive as a measure of experimentally induced central sensitization.

### Limitations and future directions

Despite benefits related to the use of laser stimulations to evoke brain responses, contact heat stimulation is less likely to cause skin burns^9^. In our study, minor skin burns were clearly evident in two subjects following laser stimulation. While noted as a common concern related to the acquisition of LEPs, their incidence is not generally reported. Another limitation of laser stimulation is the obvious need for eye protection and special room design considerations (e.g., protective barriers). Recent studies have reported using a commercially available contact heat stimulation device capable of achieving 300 °C/s, which is nominally ~ 3 times faster than currently available stimulators^22,55^. Based on our results, such technological advances should improve the acquisition of CHEPs, potentially yielding CHEPs comparable in size and latency to that acquired in response to laser stimulation. Future studies are warranted to investigate if new contact heat stimulators deliver “laser-like” evoked potentials, such as the recently developed thermal cutaneous stimulator (TCS, QST.lab)^22^. Additionally, single trial analysis, as we recently applied for CHEPs^56^, could provide insights as to whether amplitude differences between CHEPs and LEPs is the result of differences in habituation. Greater habituation might be expected for contact heat stimulation, owing to a larger stimulation surface compared to laser. Other factors, like mood, hunger, menstrual cycle etc. could have potentially influences our CHEPs and LEPs. However, given our study design it was difficult to control for those factors.

Another limitation concerned relates to our use of topical capsaicin. Although there was a significant increase in mechanical hyperalgesia, it was still small (less than 1 on the NRS overall). Also, some subjects did not respond to capsaicin. A more pronounced area of secondary hyperalgesia could potentially indicate if one method is more suitable than the other to detect sensitization. Additionally, it would have been beneficial to also have a condition with no intervention (no cream application), because the behavioural and cortical response to sham treatment is different between subjects^57^. However, adding another condition would have made the study even more comprehensive.

## Conclusion

In summary, after adjusting for perceived pain intensity, laser stimulation yielded larger cortical responses occurring at shorter latency compared to contact heat. The induction of central sensitization with capsaicin did not affect either LEPs or CHEPs, thereby providing evidence that type II A-delta mechano-heat nociceptors are not involved generating secondary hyperalgesia.

## Supplementary Information

Supplementary Information.
