# Supplementary material for: An intensity matched comparison of laser- and contact heat evoked potentials
Source: Sci Rep. 2021 Mar 25;11:6861. doi: 10.1038/s41598-021-85819-w (PMC7994633; doi:10.1038/s41598-021-85819-w)
Supplement: Supplementary file 1 — Supplementary Information. [file 41598_2021_85819_MOESM1_ESM.pdf]

An Intensity Matched Comparison of Laser- and Contact Heat Evoked Potentials

Iara De Schoenmacker[1,2], Carson Berry[1], Jean-Sébastien Blouin PhD[3], Jan Rosner[2,4] MD, Michèle Hubli[2] PhD, Catherine R. Jutzeler PhD[1,3,5], John L.K. Kramer PhD[1,3]*

[1]International Collaboration on Repair Discoveries (ICORD), University of British Columbia, Vancouver, BC, Canada
[2]Spinal Cord Injury Center, University Hospital Balgrist, University of Zurich, Zurich, Switzerland
[3]School of Kinesiology, University of British Columbia, Vancouver, Canada
[4]Department of Neurology, University Hospital Bern, Inselspital, University of Bern, Bern, Switzerland
[5]Department of Biosystems Science and Engineering, ETH Zurich, Basel, Switzerland

Target Journal: Scientific Reports

*Corresponding Author:
Dr. John Kramer
University of British Columbia
Blusson Spinal Cord Centre
818 West 10th Avenue, Vancouver, BC, Canada, V5Z 1M9
Email: kramer@icord.org
Phone: 604.675.8876
Fax: N/A

Email addresses of other authors:
Iara De Schoenmacker: iara.deschoenmacker@balgrist.ch
Carson Berry: carson.j.berry@gmail.com
Jean-Sébastien Blouin: jsblouin@mail.ubc.ca
Jan Rosner: jan.rosner@balgrist.ch
Michèle Hubli: michele.hubli@balgrist.ch
Catherine Jutzeler: Catherine.Jutzeler@bsse.ethz.ch

TABLE S.1 A: RELEVANT RAW DATA OF THE CHEPS SESSIONS

Contact Heat Stimulation

			Post – Pre-Cream Pin Prick Pain [NRS]		Mean Contact Heat Pain [NRS]		Contact Heat Evoked Potentials									
Subject	Sex	Age Group [y]	Sham	Capsaicin	Sham	Capsaicin	Sham N1 Latency [ms]	Sham N1 Amplitude [µV]	Sham N2 Latency [ms]	Sham N2 Amplitude [µV]	Sham P2 Amplitude [µV]	Capsaicin N1 Latency [ms]	Capsaicin N1 Amplitude [µV]	Capsaicin N2 Latency [ms]	Capsaicin N2 Amplitude [µV]	Capsaicin P2 Amplitude [µV]
**C01**	F	20-25	0.2	-0.1	3.6	2.1	321	-5.9	372	-22.6	24.3	336	-6.8	374	-16.7	38.6
**C02**	F	20-25	-0.3	0.1	7.1	6.9	316	-5.1	361	-5.7	5.9	320	-4.8	363	-5.4	8.2
**C03**	F	20-25	0.0	0.2	2.5	1.5		0.0	432	-15.3	11.5		0.0	418	-13.3	18.1
**C04**	F	20-25	0.1	0.7	1.4	2.0		0.0	370	-2.7	7.1		0.0	324	-7.2	19.8
**C05**	M	36-40	0.0	0.2	1.0	1.3	379	-3.2	443	-5.4	10.1		0.0	434	-10.6	8.6
**C06**	M	26-30	0.0	0.0	3.7	1.1	354	-5.6	405	-9.1	9.2		0.0	380	-6.8	10.6
**C07**	F	26-30	0.0	0.1	3.3	2.9	364	-5.1	394	-2.5	33.8	356	-4.8	376	-10.8	24.3
**C08**	M	>40														
**C09**	M	26-30	0.1		2.1											
**C10**	M	26-30	-0.2	-0.2	6.4	5.6	380	-5.4	395	-8.7	12.2	377	-3.6	416	-5.9	16.5
**C11**	M	26-30	-0.3	-0.2	2.8	2.5	362	-3.5	399	-14.9	17.1	354	-4.2	409	-16.2	14.6
**C12**	M	20-25	0.0	0.0	2.9	1.9	359	-2.0	404	-10.1	7.6	357	-1.7	409	-5.7	10.9
**C13**	M	26-30	-0.5	-0.2	1.7	2.5	333	-2.0	377	-10.6	13.3	313	-1.8	380	-15.0	19.1
**C14**	M	26-30	0.1	0.2	2.8	2.8		0.0	495	-3.3	7	416	-3.2	422	-3.7	2.3
**C15**	F	26-30	-0.1	0.2	3.3	2.9	327	-5.6	383	-16.3	10.3	336	-1.4	395	-7.7	7.1
**C16**	F	20-25	0.0	0.0	0.7	0.7	315	-8.1	361	-22.5	13.8	305	-6.9	332	-18.4	12.8
**C17**	F	20-25	-0.3	0.1	4.4	6.1	290	-5.9	351	-18.1	23.5	316	-7.0	337	-15.4	22.8
**C18**	M	31-35	-0.1	0.2	3.5	3.0	269	-4.0	337	-7.4	10.5		0.0	391	-4.8	14.8
**C19**	M	26-30	0.0	0.1	1.8	1.6		0.0	390	-6.8	9.6	371	-8.4	404	-10.2	9.1
**C20**	M	31-35	0.0	0.0	2.9	2.2	329	-3.2	391	-9.4	7.1	340	-4.8	400	-10.8	8.9

TABLE S.2 B: RELEVANT RAW DATA OF THE LEPS SESSIONS

Laser Stimulation

	Post – Pre-Cream Pin Prick Pain [NRS]		Mean Laser Pain [NRS]		Laser Evoked Potentials										Matched Laser Intensity [J]	
Subject	Sham	Capsaicin	Sham	Capsaicin	Sham N1 Latency [ms]	Sham N1 Amplitude [µV]	Sham N2 Latency [ms]	Sham N2 Amplitude [µV]	Sham P2 Amplitude [µV]	Capsaicin N1 Latency [ms]	Capsaicin N1 Amplitude [µV]	Capsaicin N2 Latency [ms]	Capsaicin N2 Amplitude [µV]	Capsaicin P2 Amplitude [µV]	Sham	Capsaicin
**C01**	0.1	0.4	0.6	0.7	204	-2.4	232	-7.9	20.1	281	0.9	291	-8.1	3.6	2.50	3.00
**C02**	-1.2	-0.6	6.2	5.5		0.0	246	-4.0	5.1		0.0	212	-3.2	2.5	2.25	2.00
**C03**	0.1	0.1	3.7	3.3	181	-11.8	195	-23.8	22.1	172	-2.1	217	-20.8	12.9	3.25	3.50
**C04**	0.1	0.2	2.4	1.2	152	-14.5	182	-12.9	10.8	177	-7.7	219	-4.9	9.2	2.50	2.75
**C05**	0.2	0.2	1.0	1.0	192	-7.2	239	-19.9	13.1	218	-6.9	249	-11.0	17.0	4.00	3.25
**C06**	-0.1	0.1	3.1	1.5	161	-1.3	203	-17.4	6.5	187	-18.6	210	-15.9	9.0	3.25	2.75
**C07**	-0.1	0.3	2.8	3.2	183	-9.2	219	-15.6	31.3	166	-8.5	188	-24.3	34.1	2.50	2.50
**C08**	-0.1	0.0	0.6	0.7											3.00	2.50
**C09**	0.1	0.1	2.5	2.4	168	-8.8	199	-19.0	13.3	147	-0.9	198	-19.5	11.5	3.00	3.25
**C10**	0.1	0.0	6.3		183	-13.5	213	-32.0	21.2	188	-14.7	217	-33.8	19.8	3.25	3.50
**C11**	0.2	-0.5	2.5	3.2	163	-3.9	224	-24.7	21.8	202	-6.5	250	-16.2	15.8	2.50	2.25
**C12**	0.0	0.0	2.4	2.2	175	-5.3	220	-19.8	17.2		0.0	222	-16.1	14.1	2.50	3.00
**C13**	-0.2	-0.2	1.3	1.8	162	-4.4	198	-9.8	15.6	153	-8.4	193	-20.4	23.9	2.50	2.75
**C14**	-0.1	0.2	3.3	3.0		0.0	202	-9.9	12.4		0.0	222	-10.0	12.3		3.00
**C15**	-0.2	0.3	4.2	3.4	156	-8.8	195	-27.6	13.5	150	-8.0	194	-30.5	15.9	3.00	
**C16**	0.0	0.0	1.5	0.4	152	-11.3	185	-31.2	25.1	161	-10.9	194	-34.6	18.1	3.25	2.75
**C17**	-0.1	0.4	3.6	5.1	149	-8.1	189	-26.9	29.6	152	-8.2	182	-30.9	35.1	4.00	2.75
**C18**	0.3	1.5	1.5	4.7	229	-3.3	244	-7.6	11.2	163	-7.0	198	-9.8	14.0	2.50	2.50
**C19**	0.0	0.1	1.0		209	-9.7	210	-18.6	18.7		0.0	221	-19.6	19.3	3.00	3.00
**C20**	0.0	0.5	4.8	2.4	159	-10.9	211	-29.9	10.7	166	-4.5	222	-22.9	11.0	3.25	2.50

DRS	# LEPs stim.	LEPs stim. Intensity [J]	LEPs NRS
1	1	1,50	0
2	2	1,50	0
2	3	2,00	1
2	4	2,25	1
1	5	2,00	1
2	6	2,50	2
1	7	2,25	2
2	8	2,75	3
1	9	2,50	2
1	10	2,75	3
2	11	2,50	2
2	12	2,25	1
1	13	2,50	3
1	14	2,25	1
1	15	2,50	2
2	16	2,50	3
2	17	2,25	1
1	18	2,75	3
2	19	2,50	2
1	20	2,50	3

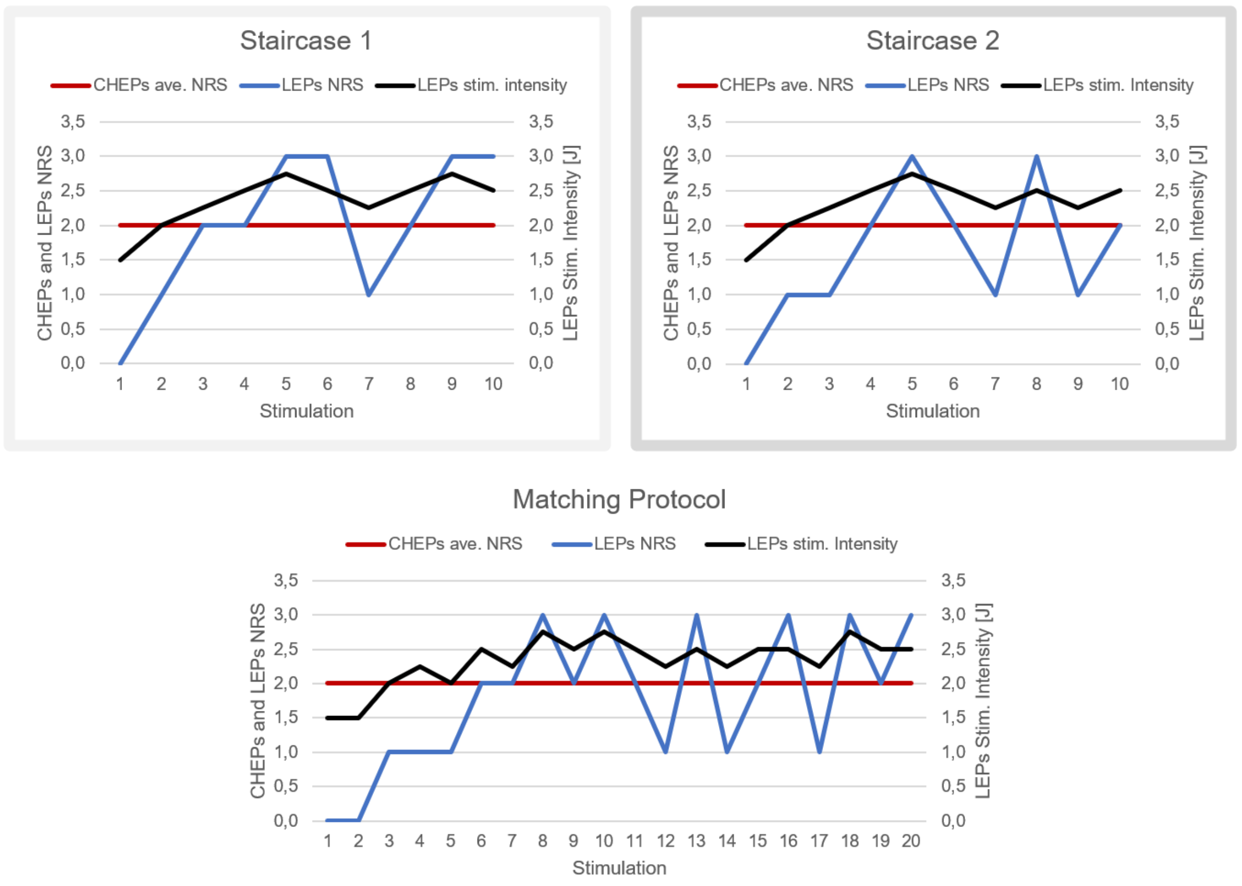

FIGURE S.1: *Double Random Staircase (DRS) Protocol. The two staircases (staircase 1: light grey, staircase 2: dark grey) of one subject are separately illustrated in the two upper graphs. The lower graph illustrates the whole matching protocol with the two staircases randomized. The red line shows the average numeric pain rating (NRS) of the 10 familiarization CHEPs. The blue and black line shows the NRS and stimulation intensity of the LEPs during the matching protocol, respectively.*
